# The transition of Chilean adolescents from the child welfare system to the adolescent justice system: a continuation or an accumulation of adverse factors?

**DOI:** 10.3389/fpsyg.2023.1194294

**Published:** 2023-07-13

**Authors:** Viviana Zambrano, Gloria Fernández-Pacheco, Miguel Salazar-Muñoz

**Affiliations:** ^1^Facultad de Derecho y Ciencias Sociales, Universidad San Sebastián, Santiago, Chile; ^2^Loyola University, Sevilla, Andalusia, Spain; ^3^Facultad de Psicología y Humanidades, Universidad San Sebastián, Santiago, Chile

**Keywords:** child welfare, juvenile justice, adolescent offender, risk factor, adverse experiences

## Abstract

Research on juvenile delinquency and adolescent maladjustment indicates that the beginning of these processes is found in the relationship between multiple risk factors at the individual, family and community levels in this population. The objective of this research was to analyze the risk factors related to the transition from the child welfare system to the adolescent justice system in a group of Chilean male adolescents (*n* = 108), aged 14–18 years, grouped according to their membership in the child welfare system, the adolescent justice system or both systems. Through a quantitative methodology, variables associated with risk factors were examined by means of the Risk and Resource Evaluation Form FER-R and the Risk and Criminogenic Needs Inventory IRNC instruments. Logistic regression analysis found that the adolescent population within the child welfare system was more likely to enter the adolescent justice system if the following risk factors were present: weak family supervision, consumption of drugs, socially maladaptive peer relationships, and risky free time. These results emphasize that child welfare system interventions should focus on parental support and the management of socio-community networks to prevent re-entry of the adolescent population into the justice system.

## 1. Introduction

There is consensus in the scientific literature regarding the relevance of promoting research on the life trajectories of the adolescent population involved in the child welfare and adolescent justice systems, since it contributes to understanding the factors that have been determining factors in the changes in the life trajectory of children and adolescents that have triggered delinquent behaviors (Moffitt, 1993; Farrington, 1995; Fréchette and LeBlanc, 1998; Loeber et al., 1998; Sampson and Laub, 2005).

In Chile, research on adolescent offenders has developed progressively in the last decade, defined by characterization studies and the generation of psychosocial technology for the development of specialized and multilevel interventions (Alarcón et al., 2017; Pérez-Luco et al., 2017; Salazar-Muñoz et al., 2020). However, within this area, there is little scientific analysis of the knowledge of risk factors and other psychosocial antecedents present in the lives of the adolescent population that transitions from the child welfare system to the adolescent justice system, an aspect that hinders the design of effective preventive programs in early stages of development (Miranda and Zambrano, 2017).

Álvarez (2011), after analyzing a government database of 20,111 adolescents who graduated from child protection programs of Chile’s National Service for Minors (Servicio Nacional de Menores de Chile, SENAME), reported that at least 7.9% of the adolescents who had graduated from these programs subsequently entered the adolescent justice system within the following 12 months. In addition, the report established that these adolescents were mostly boys and presented the following characteristics: (i) disruptive behaviors in the child welfare system; (ii) very low or very prolonged permanence in the child welfare system; (iii) runaways from residential centers; (iv) failure to meet objectives at the end of the individual intervention plan; (v) absenteeism or disengagement from school at the time of discharge; and (vi) no defined place of discharge. The same author, after analyzing a different cohort of child protection program graduates, found similar results, concluding that one of the main aspects to be reinforced in the child welfare system is the effectiveness of the interventions, since in both studies the results suggest that the transit of adolescents through the child welfare system increased the risks of developing pro-delinquent behavior in adolescence (Álvarez, 2014).

The data reported above tend to coincide with those described in various international studies, which have related the passage of adolescents through the child welfare system to a negative impact on their development (Traube et al., 2012; Barrett et al., 2014; Shpiegel, 2016; Hall et al., 2018) and increased likelihood of involvement in problems with the justice system (Baskin and Sommers, 2011; Goodkind et al., 2013). It has been estimated that more than one-third of adolescents who have been in the child welfare systems at later stages, even as little as months after leaving the system, are part of the justice systems (Dannerbeck and Yan, 2011; Lee and Villagrana, 2015; Yang et al., 2017). These figures reinforce the importance of research in this area in order to promote preventive interventions to interrupt the emergence of criminal behavior among adolescents who have experienced rights violations during their childhood and/or adolescence.

### 1.1. Adverse life experiences and adolescent maladjustment

While there is no linearity between the presence of adverse life experiences in childhood and the presence of transgressive behavior in adolescence, much of the literature that has addressed the study of adolescent maladjustment tends to indicate that a high accumulation of adverse experiences in childhood may be associated with severe patterns of offending in later life (Duke et al., 2010). Most of the findings point to the fact that victimization trajectories in adolescents who have been part of child welfare systems are marked by adverse experiences related to social disadvantage, school disengagement, drug use, unsuccessful interventions, child maltreatment and abuse, that is, factors that in some research have been found to be predictors of the onset of criminal careers (Álvarez, 2014; Gonzalez-Blanks and Yates, 2015; Yang et al., 2021; Pires and Almeida, 2023). In that vein, other research examining the relationship between adverse experiences and adolescent offending yields discouraging results, reporting that 96% of adolescent offenders have had at least one adverse experience in their life history and at least 40% have experienced four or more (Baglivio et al., 2014, 2015; Fox et al., 2015; Wolff et al., 2017).

Regarding the consequences of institutionalization in childhood, it has been found that the institutional environment has been associated with problems of altered development in children (Smyke et al., 2010). This is due to the fact that institutionalization is considered a high-stress situation, which has a negative impact on the lives of children and adolescents, given that in many cases it accentuates cognitive deterioration, social deficits, emotional alterations, aggressiveness, delinquency and isolation in this population (Fernández-Daza and Fernández-Parra, 2013).

Among the factors mentioned so far, it has been pointed out that child maltreatment is the main factor that crosses the trajectories of the adolescent population that transits from child welfare programs to the justice systems (Yang et al., 2021). In general, research indicates that many children who have experienced situations in which they have been victims of sexual abuse, physical abuse, and parental neglect appear to be more likely to enter criminal careers (Gonzalez-Blanks and Yates, 2015; Braga et al., 2017; Doelman et al., 2021). These studies report that family history of abuse and neglect increases the risk of arrest by 55% and the likelihood of committing a violent crime by 96% (Anda et al., 2010; Halemba and Siegel, 2011). These statistics show that this type of adverse experience has a considerable negative impact on the lives of children and adolescents (Danese and McEwen, 2012; Cicchetti, 2013; Baglivio et al., 2014).

It is worth noting that most of these traumatic experiences have been mostly explored in children and adolescents within a community (Humphrey and van Brunschot, 2018), not so within the child welfare system. Research with minors who are part of the child welfare system is still scarce both nationally and internationally. However, in this direction, research such as that carried out by authors such as Yampolskaya et al. (2011) and Malvaso et al. (2018), who developed studies in which they concluded that persistent maltreatment was a mediating factor in the occurrence of violent behaviors and that each report of maltreatment was associated with a 10% increase in the likelihood of being referred to child welfare, and a 15% increase in the likelihood of being detained in an adolescent justice center for delinquent offenses.

### 1.2. Transition from the child welfare system to the adolescent justice system

In view of the empirical evidence, it is clear that there is a relationship between the chronicity of abuse in the family environment and the initiation of criminal behavior by adolescents, which is mediated by the presence of other risk factors at the individual, family and community levels (Valverde, 2002).

In this field of research, Mayorga et al. (2020) analyzed a sample of 450 adolescents comprised of adolescent offenders, adolescents at risk of committing delinquent acts who had entered the child welfare system and the general adolescent population. Their results are consistent with previous research, as they indicate that adolescents at risk of delinquency and of entering the adolescent justice system present greater individual maladjustment at the personal and family levels compared to the general population. It is noteworthy that among the results of this study, the group at risk of delinquency that had entered the child welfare system and the group of lawbreakers presented deficits in common in different areas, suggesting that the level of risk of the first group to transit to the adolescent justice system is high.

Similarly, a significant amount of research has suggested that the relationship between certain individual factors, parental styles, histories of abuse, adverse experiences and environments in which adolescents are disadvantaged or vulnerable are high-risk factors for the consolidation of delinquent behaviors in this age group (Sallés and Ger, 2011; Sánchez-Teruel, 2012). One of these individual factors that has been widely documented is related to mental health, and it has been estimated that between 40 and 70% of the adolescent population that has entered the justice system has diagnosed mental health problems (Zhang et al., 2011; Barrett et al., 2014); however, although the percentages are high, it is not possible to affirm that the presence of a mental illness is the cause or predictor of delinquency (Braga et al., 2017).

In view of these factors, education is considered a protective factor par excellence when dealing with the vulnerable adolescent population, as pointed out by some studies such as the one conducted by Mersky et al. (2012), which establishes that both safety and school participation act as protective factors in preventing young people from participating in the commission of violent crimes. This has been corroborated in more recent studies such as the one conducted by Lee and Villagrana (2015), where education is recognized as an important domain that should be strengthened in the work with the adolescent population that is part of the child welfare system in order to prevent involvement in crime.

In relation to problematic substance use, the literature has shown that the adolescent population that is part of the child welfare system has a higher probability of having problematic use and at an earlier age (Cheng and Lo, 2011; Traube et al., 2012; Maneiro et al., 2016; Shpiegel, 2016). On these findings, some studies point to coping mechanisms and/or exposure from before birth to parental consumption as risk factors for problematic consumption (Dannerbeck and Yan, 2011; Fox et al., 2015). In this sense, the literature finds that substance use is not predictive of an increase in delinquency; rather, there is a relationship of “reciprocal potentiation” (Redondo, 2008, 2012), being that these factors feed back each other and potentiate in marginal environments (Traube et al., 2012; Shpiegel, 2016).

Another risk factor considered in the scientific literature is the association with criminal peers, as it has been proven that the adolescent population that is part of the child welfare system is at greater risk of becoming involved with people with criminal behavior (Yang et al., 2021). This has been evidenced in the results of research conducted in different countries in America and Europe, where the study of the influence that the peer group may have on the participation in criminal situations by young people has been widely addressed (Pyrooz et al., 2014; Marshall et al., 2015; Yang et al., 2021).

Finally, another line of research has studied the impact that the type of parenting received can have on adolescent delinquency. The results tend to indicate that an inadequate parental style, either too permissive or too authoritarian, is a factor that contributes to the deterioration of harmonious relationships in social interaction and to delinquency-related problems in this population (Tyler and Melander, 2010). This specific factor together with other risk factors, particularly exposure to acts of violence in the family, generates an increased risk of incurring in violent behaviors, developing antisocial behaviors, committing theft and presenting problems of drug abuse (Maneiro et al., 2016); as it has been corroborated that less vigilance by parental figures or inadequate supervision by caregivers is linked to a higher risk of adolescents engaging in delinquent behaviors (Tyler and Melander, 2010).

In accordance with the above, the aim of this research was to analyze the risk factors related to the transition of adolescents admitted to the child welfare system to the adolescent justice system in the Los Ríos region, Chile. It should be noted that by identifying the main risk factors, the interventions carried out in the child welfare system could be improved, in addition to optimizing social resources and orienting them toward prevention.

## 2. Manuscript formatting

### 2.1. Methodology

A quantitative methodology was used to analyze the data collected through the Risk and Resources Evaluation Form FER-R (Alarcón, 2001) and the Inventory of Risk and Criminogenic Needs IRNC (Chesta, 2009), validated in the Chilean adolescent population (Alarcón, 2001; Alarcón et al., 2009, 2022).

The research method used in the study sought to test the following hypotheses:

*H1*: The greater number of risk factors present in adolescents (weak parental supervision, maladaptive peer relationships, school disengagement, drug use, leisure time and personality difficulties), the more likelihood of moving from the child welfare system to the adolescent justice system.

*H0*: A higher number of adolescent risk factors (weak parental supervision, maladaptive peer relationships, school disengagement, drug use, leisure time and personality difficulties) does not increase the likelihood of moving from the child welfare system to the adolescent justice system.

### 2.2. Participants

Data were collected following the necessary ethical protocols. In compliance with these, first, the approval of the Ethics Committee of Loyola University was obtained and, subsequently, at the time of collecting the data necessary for the study, formal authorization was requested from SENAME, the public body in charge of administering the child welfare and adolescent justice system in Chile. Data collection took place between November 2019 and January 2020. Prior to the application of the instruments, informed consent forms were signed by the participants.

The study sample was made up of the adolescent population residing in the Los Ríos Region (Chile), users of SENAME and aged between 14 and 18 years old. Due to the low representation of women in the adolescent justice system, it was decided to exclude the female adolescent population from the final sample. It was also decided to exclude from the sample all children under 14 years of age who are part of the child welfare system, with the objective of forming homogeneous groups in relation to age for the study.

After applying the two exclusion criteria mentioned above and discarding those questionnaires that were not completed by the adolescents, the final sample consisted of *n* = 108 male adolescents. This sample was differentiated into three groups:

1. Child welfare trajectory: this group consisted of *n* = 21 adolescents who were part of child welfare programs and had no history of involvement with the adolescent justice system. The average age of the adolescents in this group was 15.7 (SD = 2.9), with a mean of 11.3 (SD = 2.9) years of schooling.
2. Justice trajectory: this group consisted of *n* = 61 adolescents who were serving sentences in open and closed settings as a result of a court conviction, and who had no history of involvement with the child welfare system. The average age of this group was 16.14 (SD = 2.7), with a mean of 9.7 years of schooling.
3. Mixed trajectory: this group consisted of *n* = 26 adolescents who are part of the adolescent justice system and had a history of being part of the child welfare system. The average age of this group was 16.2 (SD = 3.1), with a mean of 10.2 years of schooling.

### 2.3. Instruments

Risk and Resource Assessment Form (Ficha de Evaluación de Riesgos y Recursos, FER-R): is a multidimensional instrument of structured professional judgment. It was created in Chile by Alarcón (2001) to assess the presence of criminogenic risks and resources in the adolescent population under criminal sanctions. The FER-R instrument is composed of 57 items, 39 of which measure criminogenic risks and the other 18 measure adaptive resources. It is scored by presence or absence, that is, it is a dichotomous response instrument. We chose to use this tool because several investigations conducted in Chile with the FER-R have reported adequate psychometric properties of the instrument (Alarcón, 2001; Alarcón et al., 2009, 2022). In addition, the FER-R is used by many teams working in the adolescent justice system in the country (Alarcón et al., 2012).

Specifically, four indices of the instrument were used for this research:

1. Weak family supervision: The five items considered correspond to the following factors: “Parents do not supervise the young person’s behaviors,” “the family appears unorganized,” “there are no delimited roles,” “inconsistent parental role” and “parents present emotional and/or social maladaptive behaviors.” By means of a simple sum, an index was obtained that ranged between 0 and 5, where higher values indicate greater weakness of parental supervision, reaching an acceptable internal consistency (α 0.74).
2. Relationship with socially maladapted peers: this was obtained by the simple sum of the following items: “The friends in the area where he/she lives do not attend the school system,” “some friends have problems with the police and have been arrested,” “he/she does not currently belong to and is not motivated to join an organized group in his/her neighborhood or community,” “among his/her friends there are no young people working or studying satisfactorily” and “the friends and the adolescent consume alcohol and/or drugs together.” The index obtained has a range from 0 to 5 where higher values indicate a higher level of socially maladaptive peer relationships with an acceptable internal consistency (α 0.79).
3. School disengagement: composed of six items, which include the following factors: “Dropping out of school for more than 1 year,” “truancy,” “dropping out of school and not doing any paid work,” “absconding from school (more than 3 absences),” “repeating a school year (more than one)” and “poor school performance (2 fails).” This index has a range from 0 to 6, where higher values indicate a higher level of disengagement from the school system with an internal consistency within the good range (α 0.87).
4. Drug use: is composed of six items, corresponding to the following factors: “There is drug abuse,” “there is alcohol abuse,” “consumes volatile solvents or base paste,” “there is addictive behavior dependent on drugs or alcohol in a group and alone,” “drug use negatively affects their behavior and functioning in general” and “presents criminal behavior associated with drug dependence.” The simple summation of these items resulted in an index with a range from 0 to 6, where higher values indicate higher drug use with good reliability (α 0.88).

The Inventory of Risk and Criminogenic Needs (IRNC): is an assessment guideline that was developed in Chile by Chesta (2009) with the aim of establishing the risks of recidivism and identifying criminogenic factors in the adolescent offender population. This instrument is composed of 42 items that are scored by presence or absence, that is, they have a dichotomous response. It is an adaptation of the Level of Service Inventory (LSI-VI) 6th version (Andrews and Bonta, 1995). For the purposes of the research, only the “Leisure time” and “Personality” indices were considered, which are described below:

1. Risky free time: this is composed of the items “does not participate in structured activities,” “could make better use of their time” and “no personal interest.” In this index, the responses obtained were added together to obtain a variable ranging from 0 to 3, where higher values indicate the presence of greater risk in the use of free time. The reliability of the index is considered acceptable (α 0.72).
2. Personality: It is composed of the items “overestimation of self,” “physical aggressiveness,” “temper tantrums,” “limited concentration,” “inability to cope with frustration,” “insufficient sense of guilt” and “verbal aggressiveness, insolence.” These indicators, by simple addition, resulted in a variable with a range of 0 to 7 with acceptable reliability (α 0.75).

### 2.4. Data analysis

Statistical analyses were conducted with R software. To analyze the eventual association between the types of trajectories experienced by the adolescent participant population and the risk and/or protective factors identified through the FER-R and IRNC instruments, two data modelling techniques were used: (i) logistic regression for dichotomous response variables such as the existence or not of experience in the child welfare system or in the adolescent justice system, and (ii) Poisson regression for the count response variables related to the frequency of use of the programs in each system. Previously, a linear correlation analysis was performed in order to detect the existence of multicollinearity problems between the independent variables.

#### 2.4.1. Variables included in the analysis

According to the role assumed by the variables in the different regression models proposed, the following dependent and independent variables were distinguished, including the variables considered as control variables.

#### 2.4.2. Dependent variables

1. Trajectory of justice: related to the contact that the adolescent has had with the adolescent justice system due to the commission of criminal conduct. In this dichotomous response variable, those who had gone through some adolescent justice system program were coded as 1 and those who had no experience in this area were coded as 0.
2. Child welfare trajectory: in this dichotomous variable, those who had gone through a child welfare system program were identified as 1 and those who had no history of entering this system were identified as 0.
3. Mixed trajectory: this variable was coded as 1 for the adolescent population identified as having gone through the justice trajectory and 0 for those who had gone through the child welfare trajectory.

Additionally, the number of programs the young people have been through in each system was used as a dependent variable for analysis. In this way, both the variable “Number of justice programs” and the variable “Number of child welfare programs” were constructed through the sum of programs in which each young person registered participation. These were quantitative variables of a discrete nature with a value range between 0 and 5.

#### 2.4.3. Independent variables

The independent variables of the study corresponded to the different indices contained in the FER-R and IRNC instruments, previously described in the description of the instruments applied in the study. Two sociodemographic variables were also incorporated into the analysis as control variables: (i) age, which ranged between 14 and 18 years, and (ii) years of schooling, which ranged between 2 and 12 years.

## 3. Results

Table 1 presents the results of the correlations between the independent variables considered in the analysis. In this respect, it is possible to appreciate that there is no perfect collinearity between any of the pairs of regressors, the highest correlation being between the variables of drug use and school disengagement. In contrast, the variable years of schooling correlates inversely with all variables except age, with which it correlates positively, but weakly. The negative correlations can be explained by the fact that years of schooling functions as a protective factor for variables describing risk factors.

**Table 1:** Correlation matrix between independent variables.

*N* = 108	Weak family supervision	Socially maladaptive peer relationships	School desengangement	Drug use	Risky free time	Personality	Age
Socially maladaptive peer relationships	0.62***	1					
School desengagement	0.65***	0.67***	1				
Drug use	0.55***	0.68***	0.73***	1			
Risky free time	0.35***	0.35***	0.26**	0.28***	1		
Personality	0.43***	0.43***	0.52***	0.53**	0.32**	1	
Age	0.22	0.39***	0.2	0.24*	0.13*	0.11	1
Years of schooling	−0.21*	−0.29***	−0.36***	−0.23***	−0.31***	−0.11**	0.22**

*p <0.05, **p <0.01, ***p <0.001.

Table 2 shows the coefficients on the trajectory type variables, using the child well-being trajectory as the reference category for the analysis. With respect to this analysis, it is worth noting, firstly, that the variable weak family supervision has a positive and statistically significant effect on the justice trajectory and also on the mixed trajectory, using the child welfare category as a reference.

**Table 2:** Multinomial logistic models on types of trajectories.

	Model 1		Model 2	
	Justice	Mixed	Justice	Mixed
(Intercepted)	2.139^***^	−0.880	33.260^***^	16.714^*^
	(0.516)	(0.691)	(8.359)	(7.964)
Weak family supervisión	1.427^**^	0.974^*^	2.182^***^	1.266^*^
	(0.480)	(0.446)	(0.607)	(0.532)
Socially maladaptive peer relationships	−1.585^***^	−0.341	−1.785^***^	−0.367
	(0.396)	(0.313)	(0.517)	(0.370)
School desengagement	0.699^*^	0.485^·^	0.754^*^	0.572^*^
	(0.284)	(0.258)	(0.338)	(0.287)
Drug use	−0.574^*^	−0.078	−0.664^*^	−0.136
	(0.284)	(0.253)	(0.338)	(0.278)
Risky free time	−0.878^*^	−0.122	−1.433^*^	−0.234
	(0.416)	(0.353)	(0.575)	(0.442)
Personality	−0.099	−0.203	−0.163	−0.235
	(0.223)	(0.216)	(0.253)	(0.231)
Age			−1.775^***^	−1.031^*^
			(0.508)	(0.493)
Years of schooling			−0.203	−0.048
			(0.226)	(0.198)
				
AIC	162.658		143.860	
BIC	200.208		192.139	
Log Likelihood	−67.329		−53.930	
Deviance	134.658		107.860	
Num. obs.	108		108	
K	3		3	

^***^p < 0.001, ^**^p < 0.01, ^*^p < 0.05, *p* < 0.1.

This suggests that weak supervision by parents or caregivers of adolescents increases the likelihood that youth will go through the adolescent justice system or both systems compared to the child welfare system. These effects maintained statistical significance when controlling for control variables (model 2).

Regarding the age variable, the results show an inverse and statistically significant relationship with the justice trajectory and also with the mixed trajectory, in other words, the younger the age, the higher the chances of entering both systems; while the variable years of schooling does not show a significant relationship with the trajectories.

On the other hand, it was found that the variables relationship with socially maladjusted peers and drug use correlate negatively with entry into the adolescent justice system, showing no significant effect on mixed trajectory. These results can be explained by the social desirability bias that operates in the responses of many adolescents who are linked to the justice system, and should therefore be analyzed with caution.

With respect to school disengagement, model 2 showed that this variable increases the likelihood of the adolescent going through the justice and mixed trajectories, compared to only going through the child welfare system.

In relation to the use of risky free time, both model 1 and model 2 indicated that the less risky free time young people have, the more likely they are to go through the justice system. Finally, with respect to the personality variable, this variable did not show significant effects on the trajectories.

Subsequently, to complete the analysis, models for count variables were estimated by applying Poisson regression, using as the dependent variable the “number of child welfare or justice programs” in which the adolescents in the sample had participated (see Table 3 for more details). With respect to the number of programs in the adolescent justice system, only two variables were found to have significant effects that withstand statistical control. According to model 2, the index of weak family supervision has a positive effect on the number of programs in the adolescent justice system (*β* = 0.212, *p* < 0.05). On the contrary, the risky free time variable has a negative effect on the length of time spent in the juvenile justice system (*β* = −0.169, *p* < 0.05); which means that the less quality free time the adolescent has, the more likely he/she is to spend more time in the juvenile justice system. In the estimation of other variables such as school disengagement, relationship with socially maladaptive peers, drug use and personality, it was found that these have no significant effect on the dependent variable.

**Table 3:** Count models (Poisson) on the number of programs in the trajectory.

	Offender		Protection	
	1	2	3	4
Intercepted	0.458^***^	2.323^*^	−1.149^***^	−5.672^***^
	(0.127)	(0.954)	(0.233)	(1.677)
Weak family supervision	0.190^*^	0.212^*^	−0.192	−0.145
	(0.085)	(0.089)	(0.128)	(0.125)
Socially maladaptive peer relationships	−0.125^·^	−0.097	0.541^***^	0.459^***^
	(0.067)	(0.071)	(0.087)	(0.090)
School desengagement	0.114^*^	0.083	0.352^*^	0.362^*^
	(0.055)	(0.058)	(0.071)	(0.074)
Drug use	−0.049	−0.046	0.198^**^	0.204^**^
	(0.058)	(0.058)	(0.072)	(0.074)
Risky free time	−0.132	−0.169^*^	0.001	−0.049
	(0.082)	(0.086)	(0.105)	(0.113)
Personality	−0.056	−0.049	−0.002	−0.007
	(0.049)	(0.050)	(0.063)	(0.066)
Age		−0.089		0.301^**^
		(0.065)		(0.107)
Years of schooling		−0.051		−0.031
		(0.038)		(0.049)
AIC	337.996	336.402	234.817	230.350
BIC	356.771	360.541	253.592	254.489
Log Likelihood	−161.998	−159.201	−110.409	−106.175
Deviance	101.641	96.046	100.321	91.854
Núm. obs.	108	108	108	108

^***^p < 0.001, ^**^p < 0.01, ^*^p < 0.05, ^·^*p* < 0.1.

In the case of the duration of trajectories in the child welfare system, model 4 showed that there are three variables whose effects are relevant. These variables are: firstly, the relationship with socially maladjusted peers, which has a positive and highly significant effect on the duration of child welfare trajectories (*β* = 0.459, *p* < 0.001); it is followed by the school disengagement variable showing a direct and significant effect on the duration of the experience in the child welfare system (*β* = 0.362, *p* < 0.05), followed, in third place, by the drug use variable, which was shown to have a direct effect on drug use (*β* = 0.204, *p* < 0.01) on the number of programs in the child welfare system in which young people have participated.

## 4. Discussion

Based on the results obtained, the risk factors identified in the adolescent population in the child welfare system that show a higher correlation with admission to the adolescent justice system are weak family supervision, relationship with maladaptive peers, drug use, and time risk free. These findings confirm the H1 for the aforementioned variables and show consistency with previous research in Chile (Álvarez, 2014) and other countries (Gonzalez-Blanks and Yates, 2015; Yang et al., 2021), where adverse experiences related to Social disadvantage, school dropout, drug use and child abuse (inadequate upbringing) have been found to be predictors of criminal career initiation.

In the context of these risk factors, education was identified as a protective factor, given that the less school disengagement there is, the less likely that the adolescent population will be to enter the child welfare system. This corroborates the scientific basis that shows that school participation and school safety reduce the involvement of young people in delinquent behavior; even more so in the case of adolescents who have had adverse life experiences related to situations of family abuse (Mersky et al., 2012; Lee and Villagrana, 2015).

On the other hand, in the group made up of adolescents entering the adolescent justice system, it was found that the risk variables are mostly related to weak family supervision and school disengagement, and with the application of the linear regression model, risky free time was added to these variables as another important risk factor. With respect to the variable weak family supervision, it was found that the presence of this variable coincides with the risk factors for the group of adolescents in the child welfare system. This result is supported by research conducted in recent years, which has shown that exposure to situations of violence in the family is a factor that can generate problems with rules and drug use in the adolescent population, as well as being a factor that could be linked to a greater number of involvement in criminal acts (Tyler and Melander, 2010; Maneiro et al., 2016). Likewise, the results point to risky use of free time and school disengagement as risk factors, which would be related to expressions of internalization and externalization and linked to adverse experiences with a considerable negative impact on developmental processes (Danese and McEwen, 2012; Cicchetti, 2013; Baglivio et al., 2014). This has been documented in published research by authors such as Braga et al. (2017), who, through a meta-analysis, concluded that adverse experiences or victimization events, such as being a victim of maltreatment, sexual abuse or family neglect, are associated with higher rates of general antisocial behavior (*r* = 0.11; 95% CI [0.08, 0.14]) and aggressive antisocial behavior (*r* = 0.11; 95% CI [0.07, 0.14]). The systematic review by Pires and Almeida (2023) corroborates the association between polyvictimization and higher rates of antisocial behavior.

It is worth stressing that although the risk factors are similar in both groups of subjects, it is important to note that having been in the child welfare system does not have a significant effect on the justice trajectory. Therefore, it is not possible to support the hypothesis that having been in the child welfare system increases the probability of initiating a criminal trajectory. In fact, the negative impact that the accumulation of adverse situations or violence can have on the initiation of criminal careers seems to have a greater impact on the initiation of criminal careers (Baglivio et al., 2014).

Weak parental supervision, school disengagement and socially maladaptive peer relationships are the risk factors which are most related to involvement in delinquent acts in both groups of adolescents. Due to the nature of the variables, there is a need to relate them to contexts of disadvantage and vulnerability associated with criminality, child maltreatment, economic hardship, various types of violence and adverse experiences (Cooley-Strickland et al., 2011; Gracia et al., 2014). Several studies have found a direct correlation between living in a community where violence is a constant occurrence (Mustaine et al., 2014) and adverse experiences as risk factors for high crime consolidation (Sallés and Ger, 2011; Sánchez-Teruel, 2012).

Despite the results obtained, there is a recognized need for further empirical research associated with risk profiles and the transition of the adolescent population from the child welfare system to the adolescent justice system. This research need has been expressed in many of the studies that have been conducted recently (Jahnukainen, 2007; Snyder and Merritt, 2014; Lee and Villagrana, 2015; Rhoades et al., 2016; Attar-Schwartz, 2017; Yang et al., 2017; Hall et al., 2018; Tordön et al., 2019), among which there is consensus that adolescents who have been through the child welfare system are more likely to have contact with the adult justice system than the general adolescent population (Jones and Lansdverk, 2006; Dannerbeck and Yan, 2011; Yang et al., 2017).

As a first limitation of the study, it is possible to mention that the correlation and regression analysis does not allow us to determine a causal relationship between the risk factors and the trajectories studied, given that the present study was carried out with a limited sample from one region of Chile. In this regard, for future research, it is suggested that a larger sample be considered that also incorporates the female adolescent population which, due to its low representativeness within the population studied, was excluded from the sample that participated in the study.

## 5. Conclusion

The main findings of this research indicate that the adolescent population institutionalized in Los Ríos region of Chile, whether in the child welfare system or in the adolescent justice system, show risk factors that tend to lead to delinquent behavior. Specifically, the risks are associated with family and social support issues, a correlation that is supported by previous scientific literature. This does not imply that the child welfare system is a predictor of the onset of adolescent offending trajectories; even though there is abundant research in the literature that has corroborated the existence of behavioral harm associated with institutionalization, and despite the fact that these studies show that in the adult justice system there is a considerable percentage of people who have been admitted to the child welfare system at some point, there is a lack of conclusive evidence to affirm that time spent in the child welfare system is indeed a predictor of the onset of delinquent behavior.

This point highlights the need to insist on preventive intervention to reduce the risk posed by the factors that promote delinquent behavior in the adolescent population with accumulated adverse experiences, rather than creating relationships between protection and offending trajectories.

Conversely, it has to be emphasized that, in Chile’s child welfare system, it is necessary to consider multi-component interventions that include the family, the educational system and the community, in addition to focusing on the exercise of positive parenting. This assessment is reached by considering that a greater openness of the system to generate links between adolescents and their families, as well as to improve school links and encourage support networks, could discourage the involvement of young people in delinquent behavior and, therefore, the beginning of offending trajectories.

Consequently, it is concluded that the findings of this study could guide future research aimed at resilience processes from an ecosystemic and gender perspective with mediating processes of overcoming adversity, which are necessary follow-up integrators in behavioral development throughout the life cycle.

Finally, for future research, it is considered relevant conduct multicenter studies of this nature to establish mechanisms to measure both the accumulated adverse experiences and the accumulated positive experiences in order to establish interventions that are more effective on the basis of well-founded knowledge. In this sense, it is considered appropriate to develop research that allows for the identification of factors that promote resilience, providing evidence of the relevance of referral and effective interventions that allow for the incorporation of the adolescent population and their families into the social context.

## Data availability statement

The raw data supporting the conclusions of this article will be made available by the authors, without undue reservation.

## Ethics statement

The studies involving human participants were reviewed and approved by Universidad Loyola Andalucia. Written informed consent to participate in this study was provided by the participants’ legal guardian/next of kin. Written informed consent was obtained from the individual(s), and minor(s)’ legal guardian/next of kin, for the publication of any potentially identifiable images or data included in this article.

## Author contributions

VZ: writing – original draft. GF-P: reviewing. MS-M: editing. All authors contributed to the article and approved the submitted version.

## Funding

This publication had the support of the Vice-Rector’s Office for Research and Doctorates of the San Sebastián University – VRID_APC23/08 Fund.

## Conflict of interest

The authors declare that the research was conducted in the absence of any commercial or financial relationships that could be construed as a potential conflict of interest.

## Publisher’s note

All claims expressed in this article are solely those of the authors and do not necessarily represent those of their affiliated organizations, or those of the publisher, the editors and the reviewers. Any product that may be evaluated in this article, or claim that may be made by its manufacturer, is not guaranteed or endorsed by the publisher.
