# Corrigendum: The transition of Chilean adolescents from the child welfare system to the adolescent justice system: a continuation or an accumulation of adverse factors?

**DOI:** 10.3389/fpsyg.2024.1462134

**Published:** 2024-07-25

**Authors:** Viviana Zambrano, Gloria Fernández-Pacheco, Miguel Salazar-Muñoz

**Affiliations:** ^1^Facultad de Derecho y Ciencias Sociales, Universidad San Sebastián, Santiago, Chile; ^2^Loyola University, Sevilla, Andalusia, Spain; ^3^Facultad de Psicología y Humanidades, Universidad San Sebastián, Santiago, Chile

**Keywords:** child welfare, juvenile justice, adolescent offender, risk factor, adverse experiences

In the published article, the Funding information was omitted in error. It was not declared that there was funding from the Universidad San Sebastian, Santiago, Chile to finance the publication. The correct Funding statement appears below.

